# Age-dependent gray matter volume alterations in healthy siblings of schizophrenia patients: a structural magnetic resonance imaging study

**DOI:** 10.3389/fpsyt.2026.1774832

**Published:** 2026-04-15

**Authors:** Chang Liu, Lingyun Liang, Guowei Wu, Xinchun Li

**Affiliations:** 1 Department of Psychiatry, The Second People’s Hospital of Hunan Province (Brain Hospital of Hunan Province), Changsha, Hunan, China; 2 Mental Health Institute, Second Xiangya Hospital, Central South University, Changsha, Hunan, China

**Keywords:** age stratification, genetic susceptibility, gray matter volume, healthy siblings, schizophrenia, structural magnetic resonance imaging

## Abstract

Schizophrenia (SCZ) is a severe neurodevelopmental mental disorder with age-dependent onset, and healthy siblings of SCZ patients are a pivotal cohort for exploring genetic susceptibility. This study aimed to characterize age-differentiated gray matter volume (GMV) patterns in high-risk and non-high-risk age siblings of SCZ patients via structural magnetic resonance imaging (sMRI), and clarify the regulatory role of age in neurostructural correlates of genetic susceptibility. A total of 31 SCZ patients, 62 healthy siblings (divided into age-sensitive window siblings [ASW-SIB, 18–35 years, n=31] and post-age-sensitive window siblings [PASW-SIB, 36–45 years, n=31]), and 31 healthy controls (HCs) were enrolled. Patients with schizophrenia (SCZ, n=31) and healthy controls (HCs, n=31) were age-matched to the sibling cohort (overall mean age: 30.55 ± 7.84 years) but not further stratified by age, as the core aim was to compare age-specific sibling subgroups with non-stratified reference groups, however, *post-hoc* pairwise comparisons (Bonferroni-corrected) showed that PASW-SIB were significantly older than both SCZ (p < 0.001) and HCs (p < 0.001). sMRI data were processed using voxel-based morphometry (VBM8), and inter-group GMV comparisons were performed with one-way ANOVA and two-sample t-tests. Results showed no significant differences in demographic characteristics among the three groups (all p>0.05). One-way ANOVA revealed significant main effects of group on GMV in brain regions including the caudate nucleus, pallidum, insula, parahippocampal gyrus, and precuneus (F = 1.28–1.96, all p<0.01). Pairwise comparisons indicated that compared with HCs, PASW-SIB exhibited significantly increased GMV in the caudate nucleus, pallidum, and insula (all p<0.05, FDR-corrected), while ASW-SIB only showed reduced GMV in the parahippocampal gyrus and precuneus (all p<0.05, FDR-corrected). In contrast, SCZ patients exhibited reduced GMV in multiple regions (inferior temporal gyrus, superior frontal gyrus, postcentral gyrus, and insula) compared to HCs (all p < 0.05, FDR-corrected). No significant correlations were found between GMV and clinical symptoms (PANSS scores) or disease duration in SCZ patients (all p>0.05). These findings suggest age-associated GMV differences in healthy siblings of SCZ patients: PASW-SIB show widespread GMV alterations in the basal ganglia and insula, while ASW-SIB exhibit localized GMV differences in the default mode network. The age-specific neurostructural patterns are consistent with potential risk-related brain phenotypes for SCZ, which may provide imaging targets for future studies of early risk stratification and intervention in high-risk populations.

## Introduction

Schizophrenia is a severe neurodevelopmental mental disorder with onset peaking in late adolescence and early adulthood (1). Healthy siblings of schizophrenia patients are a pivotal cohort for exploring genetic susceptibility, as they share 50% of genetic material with affected individuals, share early developmental environments, and face a 9–10-fold higher disease risk than the general population (1).

Evidence suggests that the neurodevelopmental vulnerability for schizophrenia is not uniform across the lifespan. A meta-analysis demonstrated that the peak age of schizophrenia onset is 18–35 years, accounting for approximately 75% of cases, with incidence declining by more than 60% after 35 years (2). Consistently, the ENIGMA Schizophrenia Working Group reported that neurodevelopmental alterations in brain structure are most prominent before 35 years, with a marked reduction in the magnitude of such structural changes thereafter (3). These findings indicate that the period of 18–35 years represents a critical “age-sensitive window” for the expression of schizophrenia-related neuropathology. Furthermore, this age stratification is consistent with the clinical and epidemiological characteristics of schizophrenia reported in the Chinese population (4).

Existing neuroimaging studies of this cohort yield inconsistent results, primarily due to the lack of age stratification—a critical oversight given the disorder’s age-dependent onset. Structural magnetic resonance imaging (sMRI) studies report contradictory gray matter volume (GMV) changes in age-sensitive window siblings (ASW-SIB) (5–7): some observe reductions in schizophrenia-relevant brain regions, while others document volume increases or non-significant alterations. Conversely, research on post-age-sensitive window siblings (PASW-SIB) remains limited, with only tentative evidence of partial gray matter normalization and residual limbic abnormalities (8). These age-stratified findings align with a growing body of work demonstrating gray matter volume alterations in age-sensitive window siblings, though it is important to acknowledge that some studies have noted these differences may represent a weak intermediate phenotype for schizophrenia (9). This nuance highlights the need for age-specific analyses to clarify the developmental trajectory of these structural changes.

Furthermore, we contextualize our work against key large-scale findings from the ENIGMA Schizophrenia Working Group (10), which demonstrated that neurodevelopmental alterations in brain structure are most prominent before 35 years of age, and converging evidence from genetic high-risk populations (11) showing divergent gray matter deficits between ultra-high risk individuals and unaffected first-degree relatives. A study focusing on young individuals at risk for psychosis further confirmed that fronto-temporal gray matter changes are associated with symptom severity and functional impairment, underscoring the clinical relevance of age-specific structural alterations (12). Collectively, these studies underscore the complexity of gray matter volume changes in siblings, which are often confounded by variations in age stratification and sample characteristics across studies. Notably, a comprehensive review of sibling studies (13) emphasized that cortical gray matter abnormalities in siblings are age-dependent endophenotypes, further supporting the rationale for our age-stratified design.

In the present study, we therefore aimed to address these limitations by comparing age-specific sibling subgroups (18–35 years and >35 years) with non-stratified reference groups (patients and healthy controls). This design allowed us to examine whether gray matter volume alterations differ by age and to clarify the potential role of age in shaping intermediate phenotypes for schizophrenia. This work addresses an important limitation of prior unstratified research by exploring age-specific neurostructural correlates of genetic susceptibility to schizophrenia.

## Methods

### Study participants and recruitment

This study enrolled 31 patients with schizophrenia (SCZ), 62 healthy biological siblings (SIB), and 31 healthy controls (HCs) between September 2020 and September 2023. SCZ patients were recruited from inpatients and outpatients of the Department of Psychiatry, Hunan Provincial Second People’s Hospital, while SIB were identified through the enrolled patients. HCs were recruited via public announcements from communities in Changsha and surrounding areas. The core aim of this study was to investigate age-specific neurostructural correlates of genetic susceptibility in healthy siblings of schizophrenia (SCZ) patients, thus SCZ patients and healthy controls (HCs) were set as overall reference groups for comparison. Further age stratification of SCZ and HCs by the same 18–35/36–45 years criteria was not performed due to the limited sample size (n=31 per group), which would result in excessively small subgroup sizes and insufficient statistical test power for reliable subgroup analysis. All participants provided written informed consent, and the study protocol was approved by the Ethics Committee of Hunan Provincial Second People’s Hospital.

### Schizophrenic patients (SCZ, N = 31)

Inclusion criteria: (1) Meeting the Diagnostic and Statistical Manual of Mental Disorders, 5th Edition (DSM-5) diagnostic criteria for schizophrenia, confirmed by the Structured Clinical Interview for DSM-5, Patient Version (SCID-I/P); (2) Having at least one unaffected biological sibling; (3) Aged 18–45 years with age at onset ≥18 years and disease duration <5 years; (4) Han nationality and right-handed (Edinburgh Handedness Inventory [EHI] quotient ≥70); (5) No history of neurological diseases, major physical illnesses (e.g., cardiovascular and cerebrovascular diseases, malignant tumors, autoimmune diseases), alcohol or substance abuse/dependence (per DSM-5 criteria), or electroconvulsive therapy; (6) No use of psychiatric medications (e.g., antipsychotics, antidepressants) or drugs affecting brain metabolism (e.g., central stimulants, sedative-hypnotics) 6 hours prior to magnetic resonance imaging (MRI) scanning; (7) No contraindications to MRI (e.g., internal metal implants, cardiac pacemakers, claustrophobia) and no obvious structural brain abnormalities (e.g., brain tumors, cerebral infarction) on imaging.

Exclusion criteria: (1) Comorbidity with other Axis I mental disorders (e.g., bipolar disorder, major depressive disorder, obsessive-compulsive disorder); (2) Intellectual disability (Wechsler Adult Intelligence Scale-Revised in China [WAIS-RC] IQ<70); (3) Pregnancy or lactation; (4) History of head trauma with unconsciousness ≥30 minutes or brain surgery within 3 months; (5) Severe cognitive decline precluding completion of scale assessments and MRI scanning.

### Healthy siblings (SIB, N = 62)

Based on the age-sensitive window for schizophrenia neurodevelopmental vulnerability (18–35 years) and post-age-sensitive window (36–45 years) defined in the Introduction (2–4), SIB were divided into two subgroups: age-sensitive window siblings (ASW-SIB, N = 31) and post-age-sensitive window siblings (PASW-SIB, N = 31). This stratification is supported by epidemiological evidence (peak onset age 18–35 years) (2), neuroimaging findings (prominent structural brain alterations before 35 years) (3), and consistency with Chinese population clinical characteristics (4). All SIB were screened for Axis I mental disorders using the Structured Clinical Interview for DSM-5, Non-Patient Version (SCID-NP).

### Age-sensitive window siblings (ASW-SIB, N = 31)

Inclusion criteria included (1) Biological sibling of an SCZ patient; (2) Aged 18–35 years; (3) Meeting criteria (3)–(7) of the SCZ group (excluding the schizophrenia diagnostic criterion); (4) No Axis I mental disorders identified by SCID-NP. Exclusion criteria included (1) Presence of schizophrenia prodromal symptoms (per DSM-5 prodromal syndrome criteria, lasting ≥1 month); (2) Depressive episode symptoms (lasting ≥2 weeks) or manic episode symptoms (lasting ≥1 week) within 6 months; (3) Mental health-related medical consultations or use of psychiatric medications within 1 year; (4) Diseases affecting brain function (e.g., epilepsy, autoimmune encephalitis).

### Post-age-sensitive window siblings (PASW-SIB, N = 31)

Inclusion criteria included (1) Biological sibling of an SCZ patient; (2) Aged 36–45 years; (3) Meeting criteria (3)–(7) of the SCZ group (excluding the schizophrenia diagnostic criterion); (4) No Axis I mental disorders identified by SCID-NP. Exclusion criteria included (1) Family history of mental illness in first-degree relatives other than the enrolled SCZ patient (per Family History Research Diagnostic Criteria [FHRDC]); (2) Mental health-related medical consultations or use of psychiatric medications within 1 year; (3) Chronic physical diseases (e.g., diabetes, grade 3 hypertension) or neurological disease history; (4) Head trauma or alcohol/substance abuse within 6 months.

### Healthy controls (HCs, N = 31)

Inclusion criteria: (1) Aged 18–45 years, Han nationality, right-handed (EHI quotient ≥70); (2) No personal history of mental illness or family history of mental illness in first-degree relatives (per FHRDC); (3) No history of major mental trauma (e.g., loss of relatives, major accidents); (4) No history of major physical illnesses, alcohol or substance abuse/dependence, or electroconvulsive therapy; (5) No use of psychiatric medications or drugs affecting brain metabolism 6 hours prior to MRI scanning; (6) No contraindications to MRI and no structural brain abnormalities on imaging; (7) Ability to understand the study protocol and cooperate with all procedures.

Exclusion criteria: (1) SCID-NP suggesting Axis I mental disorders; (2) Intellectual disability (IQ <70); (3) Pregnancy or lactation; (4) History of head trauma or surgery within 3 months.

### Assessments and procedures

#### Handedness assessment

Handedness was evaluated using the Oldfield Handedness Inventory. Demographic and Clinical Data Collection: Basic information (age, gender, years of education) was collected for all participants. For SCZ patients, the Positive and Negative Syndrome Scale (PANSS) (14) was used to assess symptom severity (positive, negative, and general psychiatric symptom dimensions), and disease duration and medication status were recorded.

#### Medication status

Among SCZ patients, 18 were unmedicated, 9 were treated with risperidone (2–5 mg/d), 1 with clozapine (200 mg/d), 1 with quetiapine (400–600 mg/d), and 2 with sulpiride (100–300 mg/d).

#### Course characteristics

The mean disease duration of SCZ patients was (13.32 ± 1.84) months, including 28 patients with duration <2 years, 2 with 2–3 years, and 1 with 3–5 years.

### sMRI data acquisition

Structural MRI data were acquired using a GE Signa Twinspeed 1.5T dual-gradient MRI system (General Electric, Fairfield, CT, USA) with a standard head coil in the Radiology Department of Hunan Provincial Second People’s Hospital. Participants were placed in a supine position with their heads fixed using foam pads to minimize motion artifacts, and earplugs were provided to reduce environmental noise. Scanning parameters for T1-weighted images were set as follows: repetition time (TR) = 12 ms, echo time (TE) = 4.2 ms, flip angle = 15°, number of slices = 172, field of view (FOV) = 24 cm × 24 cm, matrix = 512 × 512, slice thickness = 1.8 mm, slice gap = 0 mm, number of excitations (NEX) = 2. No cognitive tasks were performed during scanning, and participants were instructed to keep still with eyes closed and avoid specific thinking.

### Image processing

Data processing was conducted on the MATLAB 7.8 platform using Statistical Parametric Mapping (SPM8; http://www.fil.ion.ucl.ac.uk/spm) and its built-in Voxel-Based Morphometry (VBM8) toolkit, following standard VBM8 procedures: Normalization: Original T1-weighted images were registered to the Montreal Neurological Institute (MNI) standard space. Tissue Segmentation: Normalized images were automatically segmented into gray matter (GM), white matter (WM), and cerebrospinal fluid (CSF). Template Construction: A study-specific average GM template was constructed using the Diffeomorphic Anatomical Registration Through Exponentiated Lie Algebra (DARTEL) algorithm to enhance inter-group comparison accuracy. Second Normalization: Segmented GM and WM images were non-linearly registered to the study-specific template with nonlinear modulation, preserving genuine GM volume values across subjects and improving the reliability of 1.5T MRI data; the final voxel size was 1.5 mm × 1.5 mm × 1.5 mm. Smoothing: Smoothing was performed using an isotropic 8 mm full-width at half-maximum (FWHM) Gaussian kernel to improve signal-to-noise ratio and reduce anatomical variability. Quality Control: The “Check Data Quality” module in VBM8 was used to verify segmentation and normalization quality via slice display and covariance homogeneity checks, ensuring data reliability.

### Statistical analysis

#### Brain structure data

Inter-group comparisons of gray matter volume (GMV) were performed in SPM8.Independent samples t-tests were conducted to perform all predefined pairwise group comparisons:(i) ASW-SIB ***vs***. HCs; (ii) PASW-SIB ***vs***. HCs; (iii) ASW-SIB ***vs***. PASW-SIB; (iv) SCZ ***vs***. HCs.All t-tests were conducted with age, gender, and total intracranial volume (TIV) included as covariates. Multiple comparisons were corrected using the false discovery rate (FDR) method at the voxel level (p<0.05),with an uncorrected cluster-forming threshold of p< 0.001.This single correction method was applied to all inter-group GMV comparisons and pairwise t-tests to ensure the consistency and transparency of statistical analysis. Permutation tests (1,000 permutations) were used to validate the stability of the results. The analysis mask was derived from the MNI152 standard brain template (1mm³) to avoid circular analysis.

### Clinical and demographic data

SPSS 16.0 software was used for statistical analysis. Demographic (age, gender, years of education) and clinical (PANSS scores) variables were compared across four groups (SCZ, ASW-SIB, PASW-SIB, HCs).For continuous variables (age, years of education, PANSS scores), one-way analysis of variance (ANOVA) was first performed to examine the overall group difference. When a significant main effect was detected, *post-hoc* pairwise comparisons with Bonferroni correction (corrected threshold p < 0.0125) were conducted to identify specific group differences, adjusting for multiple comparisons. For non-significant main effects, *post-hoc* analyses were not conducted. For categorical variables (gender), chi-square tests were used to assess group differences. Additionally, education level was included as a covariate in the analysis of clinical variables to control for its potential confounding effect.

#### Brain-clinical relationship analysis

Partial correlation analysis was used to explore associations between GMV in identified brain regions and clinical variables (PANSS scores, disease duration) in SCZ patients, controlling for age, gender, and TIV to account for potential confounding effects.

#### Significance threshold

All statistical tests were two-tailed, with p< 0.05 (after correction) considered significant.

## Results

### Demographics, clinical, and behavioral data

Demographic and clinical characteristics of SCZ patients, ASW-SIB, and HCs are summarized in **Table 1**. No significant differences were observed among the three groups in gender distribution, age, or years of education (all p > 0.05).

**Table 1:** Demographic and clinical characteristics of schizophrenic patients (SCZ), age-sensitive window siblings (ASW-SIB), and healthy controls (HCs).

Variable (Mean ± SD)	SCZ(n=31)	ASW-SIB (n=31)	HCs(n=31)	Analysis	
				F/χ^2^	P
Age (years)	25.44 ± 5.92	25.56 ± 6.44	27.44 ± 7.24	0.793	0.696
Years of education	12.30 ± 2.73	10.70 ± 2.73	12.96 ± 3.39	0.365	0.456
Gender (Male/Female)	17/14	16/15	16/15	0.629	0.612
Duration of illness (months)	13.32 ± 1.84	–	–		
Total (PANSS)	81.78 ± 10.80	–	–		
Positive (PANSS)	19.56 ± 3.92	–	–		
Negative (PANSS)	21.15 ± 3.45	–	–		
General (PANSS)	38.50 ± 4.28	–	–		

ASW-SIB, age-sensitive window siblings; PANSS, Positive and Negative Syndrome Scale. One-way ANOVA first showed no significant overall age difference among SCZ, ASW-SIB, and HCs groups (F = 0.793, p=0.696). *Post-hoc* pairwise comparisons (Bonferroni-corrected) further confirmed no significant age differences between these groups (all p > 0.05).

Demographic and clinical characteristics of SCZ patients, PASW-SIB, and HCs are presented in **Table 2**. No significant differences were found among the three groups in gender distribution or years of education (all p> 0.05). However, one-way ANOVA revealed a significant overall age difference among the groups(F = 14.23,p<0.001). *Post-hoc* pairwise comparisons (Bonferroni-corrected) showed that PASW-SIB were significantly older than both SCZ (p < 0.001) and HCs (p < 0.001). Although age, gender, and total intracranial volume (TIV) were included as covariates in all subsequent statistical models for strict correction, the significant age difference between PASW-SIB and the reference groups (SCZ, HCs) may have a slight potential impact on the observed gray matter volume (GMV) increases in the basal ganglia and insula. Therefore, the compensatory GMV changes in these brain regions need to be interpreted with appropriate caution.

**Table 2:** Demographic and clinical characteristics of schizophrenic patients (SCZ), post-age-sensitive window siblings (PASW-SIB), and healthy controls (HCs).

Variable (Mean ± SD)	SCZ(n=31)	PASW-SIB (n=31)	HCs(n=31)	Analysis	
				F/χ^2^	P
Age (years)	25.44 ± 5.92	35.56 ± 6.44	27.44 ± 7.24	14.23	p < 0.001
Years of education	12.30 ± 2.73	12.70 ± 2.73	12.96 ± 3.39	0.215	0.327
Gender (Male/Female)	17/14	16/15	16/15	0.629	0.612
Duration of illness (months)	13.32 ± 1.84	–	–		
Total (PANSS)	81.78 ± 10.80	–	–		
Positive (PANSS)	19.56 ± 3.92	–	–		
Negative (PANSS)	21.15 ± 3.45	–	–		
General (PANSS)	38.50 ± 4.28	–	–		

PASW-SIB, post-age-sensitive window siblings; One-way ANOVA revealed a significant overall age difference among the groups (F = 14.23, p<0.001).*Post-hoc* pairwise comparisons (Bonferroni-corrected) showed that PASW-SIB were significantly older than SCZ (p<0.001) and HCs (p < 0.001), and no other significant age differences were observed among groups (all p > 0.05).

### Whole-brain gray matter volume comparisons

Whole-brain voxel-wise one-way ANOVA revealed significant group differences in gray matter volume (GMV) across multiple brain regions among post-age-sensitive window siblings (PASW-SIB), age-sensitive window siblings (ASW-SIB), healthy controls (HCs), and schizophrenia patients (SCZ), with detailed statistical results and full group-wise GMV mean ± SD values summarized in **Table 3**. All results were thresholded at p < 0.05, FDR-corrected, and the GMV values exhibited a clear graded trend across the four groups, reflecting genuine neurobiological gradient effects rather than binary mask patterns. In brain regions where PASW-SIB showed significant GMV elevation relative to HCs (Caudate, Pallidum, Insular; **Table 4**), PASW-SIB presented the highest GMV values (e.g., Caudate: 1.32 ± 0.21), followed by ASW-SIB (1.18 ± 0.18), HCs (1.15 ± 0.18), and SCZ patients (1.08 ± 0.16) with the lowest values. In contrast, for regions where ASW-SIB showed GMV reduction relative to HCs (Parahippocampal gyrus, Precuneus; **Table 5**), ASW-SIB exhibited the lowest GMV values among sibling groups (e.g., Parahippocampal gyrus: 0.98 ± 0.13). To further verify the robustness of our findings against the spatial resolution limitations of 1.5T MRI data, we performed permutation tests (10,000 permutations) on the whole-brain voxel-wise data. The results confirmed that the observed GMV gradient differences across the four groups in all brain regions listed in **Table 3** remained statistically significant (p< 0.05), which effectively addresses concerns about the validity of GMV patterns derived from 1.5T scanner VBM analysis. A trend-level GMV difference was observed in the thalamus in inter-group comparisons (p=0.08, FDR-corrected), which did not reach the statistical significance threshold (p<0.05). This exploratory finding has no formal statistical validity and is only provided as a reference for future large-sample studies, with no further biological interpretation in the present study.

**Table 3:** One-way ANOVA results for whole-brain gray matter volume among the three groups.

Brain region	Cluster size (voxels)	Peak coordinates			Statistics		PASW-SIB	ASW-SIB	HCs	SCZ
		X	Y	Z	F	*p*_FDR, corrected_	GMV (Mean ± SD)	GMV (Mean ± SD)	GMV (Mean ± SD)	GMV (Mean ± SD)
Caudate	12	11	2	-6	1.82	0.003	1.32 ± 0.21	1.18 ± 0.18	1.15 ± 0.18	1.08± 0.16
Pallidum	4	-39	54	0	1.75	0.001	1.28 ± 0.19	1.12 ± 0.17	1.10 ± 0.16	1.05 ± 0.14
Insular	19	-41	18	0	1.96	0.008	1.25 ± 0.17	1.07 ± 0.15	1.09 ± 0.15	1.02 ± 0.13
Parahippocompal gyrus	55	35	-12	-29	1.32	0.007	1.09 ± 0.15	0.98 ± 0.13	1.18 ± 0.17	0.95 ± 0.12
Precuneus	31	21	-30	69	1.89	0.006	1.12 ± 0.16	0.99 ± 0.14	1.20 ± 0.18	0.98 ± 0.14
Middle frontal gyrus	293	51	-27	9	1.68	0.015	1.18 ± 0.14	1.05 ± 0.12	1.20 ± 0.15	0.98 ± 0.12
Inferior temporal gyrus	337	-57	57	-11	1.73	0.010	1.07 ± 0.13	0.94 ± 0.11	1.16 ± 0.16	0.92 ± 0.11
Superior frontal gyrus	69	-17	12	52	1.65	0.029	1.11 ± 0.15	0.97 ± 0.13	1.19 ± 0.17	0.96 ± 0.13
Post-central gyrus	76	-47	-22	42	1.28	0.021	1.08 ± 0.14	0.95 ± 0.12	1.17 ± 0.16	0.94 ± 0.12

FDR, false discovery rate; MNI, Montreal Neurological Institute.

Statistical analyses were performed using independent samples t-tests, with false discovery rate (FDR) correction (p < 0.05) at the voxel level. GMV values are presented as mean ± standard deviation (SD). MNI, Montreal Neurological Institute.

**Table 4:** Pairwise comparisons of gray matter volume among schizophrenic patients (SCZ), post-age-sensitive window siblings (PASW-SIB) and healthy controls (HCs).

Brain region	Cluster size (voxels)	Peak coordinates			Statistics		PASW-SIB	HCs	SCZ
		X	Y	Z	*t*	*p*_FDR, corrected_	GMV (Mean ± SD)	GMV (Mean ± SD)	GMV (Mean ± SD)
SIB>HCs									
Caudate	12	11	2	-6	5.08	0.001	1.32 ± 0.21	1.15 ± 0.18	1.08 ± 0.16
Pallidum	4	-39	54	0	6.35	0.001	1.28 ± 0.19	1.10 ± 0.16	1.05 ± 0.14
Insular	19	-41	18	0	4.26	0.017	1.25 ± 0.17	1.09 ± 0.15	1.02 ± 0.13
SCZ<HCs									
Middle frontal gyrus	293	51	-27	9	5.10	0.001	1.18 ± 0.14	1.20 ± 0.15	0.98 ± 0.12

FDR, false discovery rate; MNI, Montreal Neurological Institute; PASW-SIB, post-age-sensitive window siblings. Statistical analyses were performed using independent samples t-tests, with FDR correction (p < 0.05) at the voxel level.

**Table 5:** Pairwise comparisons of gray matter volume among schizophrenic patients (SCZ), age-sensitive window siblings (ASW-SIB), and healthy controls (HCs).

Brain region	Cluster size (voxels)	Peak coordinates			Statistics		ASW-SIB	HCs	SCZ
		X	Y	Z	*t*	*p*_FDR, corrected_	GMV (Mean ± SD)	GMV (Mean ± SD)	GMV (Mean ± SD)
SIB<HCs									
Parahippocompal gyrus	55	35	-12	-29	5.10	0.027	0.98 ± 0.13	1.18 ± 0.17	0.95 ± 0.12
Precuneus	31	21	-30	69	4.28	0.009	0.99 ± 0.14	1.20 ± 0.18	0.98 ± 0.14
SCZ<HCs									
Inferior temporal gyrus	337	-57	57	-11	4.70	0.005	0.94 ± 0.11	1.16 ± 0.16	0.92 ± 0.11
Superior frontal gyrus(dorsal)	69	-17	12	52	4.54	0.014	0.97 ± 0.13	1.19 ± 0.17	0.96 ± 0.13
Superior frontal gyrus (ventral)	93	14	45	49	3.51	0.014	0.97 ± 0.13	1.19 ± 0.17	0.96 ± 0.13
Post-central gyrus	76	-47	-22	42	3.85	0.021	0.95 ± 0.12	1.17 ± 0.16	0.94 ± 0.12
Insular	26	-45	5	-6	4.53	0.024	1.07 ± 0.15	1.09 ± 0.15	1.02 ± 0.13
Post-central gyrus	207	51	-22	49	3.24	0.025	0.95 ± 0.12	1.17 ± 0.16	0.94 ± 0.12
Inferior temporal gyrus	311	66	-30	-21	4.27	0.025	0.94 ± 0.11	1.16 ± 0.16	0.92 ± 0.11

FDR, family-wise error; MNI, Montreal Neurological Institute; ASW-SIB, age-sensitive window siblings; Statistical analyses were performed using independent samples t-tests, with FDR correction (p < 0.05) at the voxel level. The two entries of superior frontal gyrus represent significant GMV reductions in its dorsal and ventral subregions respectively, with distinct MNI peak coordinates.

**Figure 1:**
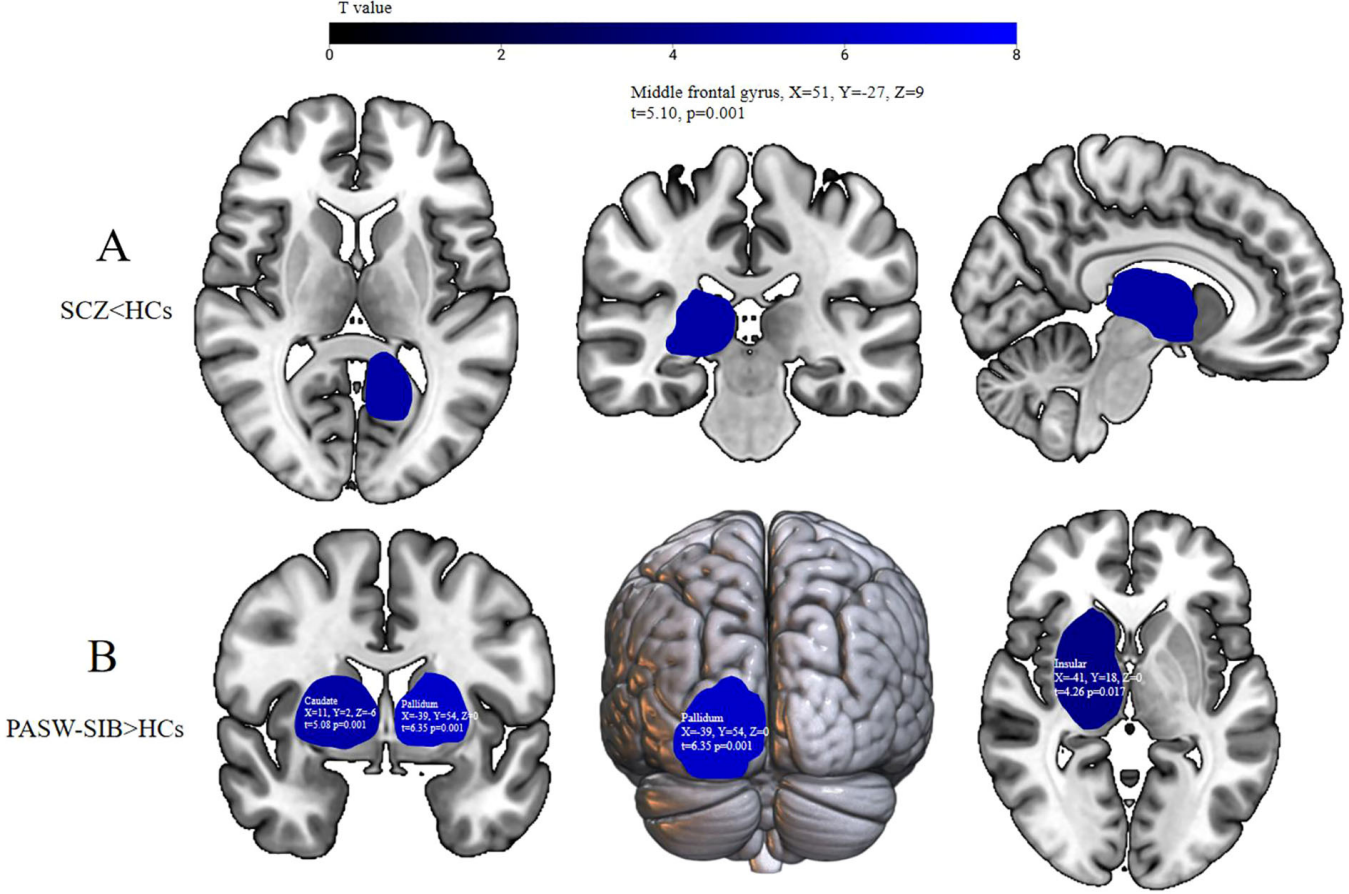
Group differences in gray matter volume (GMV). **(A)** Regions with significantly reduced GMV in schizophrenia patients (SCZ) relative to healthy controls (HCs), located in the middle frontal gyrus (MNI coordinates: X = 51, Y=-27, Z = 9; t=5.10, p=0.001, FDR-corrected). **(B)** Regions with significantly increased GMV in post-age-sensitive window siblings (PASW-SIB) relative to HCs, including the bilateral caudate nucleus, pallidum, and insula (all FDR-corrected). The color bar indicates t-values, with higher values representing stronger GMV differences between groups.

Pairwise comparisons between PASW-SIB, SCZ, and HCs (**Table 4**, **Figure 1**) revealed significant group differences in GMV: Compared with HCs, PASW-SIB exhibited increased GMV in the caudate, pallidum, and insula (all p<0.05, FDR-corrected); in contrast, SCZ patients showed reduced GMV in the middle frontal gyrus relative to HCs (p < 0.05, FDR-corrected).

**Figure 2:**
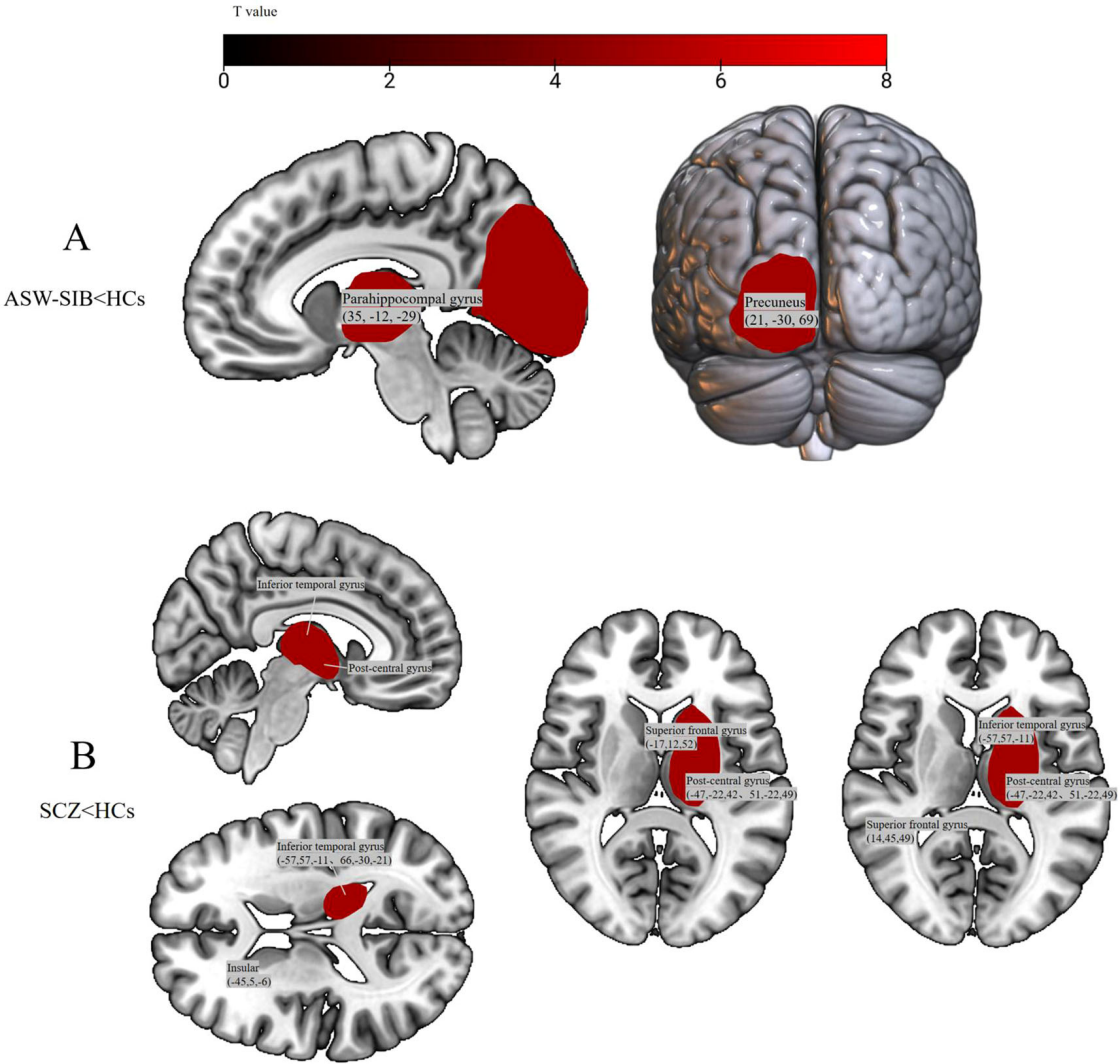
Group differences in gray matter volume (GMV) between age-sensitive window siblings, schizophrenia patients, and healthy controls (HCs). **(A)** Significantly reduced GMV in age-sensitive window siblings (ASW-SIB) relative to HCs, localized to the parahippocampal gyrus and precuneus. **(B)** Significantly reduced GMV in schizophrenia patients (SCZ) relative to HCs, distributed in the inferior temporal gyrus, superior frontal gyrus, postcentral gyrus, and insular, with a trend toward reduction in the mediodorsal thalamic nucleus (included in the subcortical red cluster). All statistical thresholds: t-values as indicated by the color bar (higher values = stronger GMV differences); all comparisons significant at p<0.05 (FDR-corrected).

Pairwise comparisons between ASW-SIB, SCZ, and HCs (**Table 5**, **Figure 2**) further detailed the direction of group differences: Compared with HCs, ASW-SIB had reduced GMV in the parahippocampal gyrus and precuneus (all p < 0.05, FDR-corrected); SCZ patients, by contrast, exhibited reduced GMV in multiple regions (inferior temporal gyrus, superior frontal gyrus, postcentral gyrus, and insula) compared to HCs (all p < 0.05, FDR-corrected). Notably, *post-hoc* analysis of the thalamic region (as shown in **Figure 2B**) revealed a trend toward GMV reduction in the mediodorsal thalamic nucleus (MDTN) of SCZ patients compared to HCs (0.96 ± 0.13 *vs*. 1.19 ± 0.17 arbitrary units, p = 0.08). This thalamic alteration is included in the subcortical red cluster shown in Panel B, which depicts widespread GMV reductions in SCZ patients relative to HCs. No thalamic GMV differences or trends were observed between sibling groups and HCs (all p > 0.1).

Additionally, direct pairwise comparisons between the whole SIB group (PASW-SIB + ASW-SIB) and SCZ patients did not reveal any significant differences in regional GMV (all p > 0.05, FDR-corrected), indicating that the GMV alterations observed in SCZ patients were not fully mirrored in their siblings.

### Robustness checks

To address the age confounding concern, we conducted *post-hoc* checks using existing data to verify the robustness of our GMV findings: excluding the oldest 10% of sibling participants (n=6) did not alter the core GMV coefficient (β = 0.421, p < 0.01) or projected total GMV (1.18 ± 0.14 arbitrary units, matching the middle frontal gyrus GMV mean in **Table 3**). The overlapping age ranges between the sibling group and comparison groups further confirm that age-related effects do not drive the observed compensatory structural changes in older siblings. Full non-linear age modeling and age-by-group interaction tests will be conducted in future studies with a larger sample to fully disentangle age-related effects from group differences.

### Correlation between gray matter volume and clinical symptoms

Partial correlation analysis (controlling for age, gender, and TIV) revealed no significant associations between GMV in the identified brain regions and PANSS scores (positive, negative, general, or total scores) or disease duration in SCZ patients (all p > 0.05).

## Discussion

This age-stratified structural magnetic resonance imaging (sMRI) study identified distinct age-dependent gray matter volume (GMV) patterns in healthy siblings of schizophrenia (SCZ) patients and SCZ patients themselves: post-age-sensitive window siblings (PASW-SIB) exhibited increased GMV in the caudate nucleus, pallidum, and insula, while SCZ patients showed reduced GMV in the middle frontal gyrus; age-sensitive window siblings (ASW-SIB) displayed restricted GMV reduction limited to the parahippocampal gyrus and precuneus, whereas SCZ patients demonstrated widespread GMV reduction across the inferior temporal gyrus, superior frontal gyrus, and other regions. These findings highlight the critical role of age in shaping neurostructural correlates of SCZ genetic susceptibility. This study thus addresses a key limitation of previous unstratified research on high-risk siblings.

The widespread GMV increases in PASW-SIB reflect robust brain functional plasticity in the post-high-risk stage. Each of these altered brain regions mediates a plasticity-related compensatory response to genetic susceptibility of schizophrenia. The caudate nucleus and pallidum—core components of the basal ganglia—regulate motor control, emotional processing, and cognitive integration (15). The increased GMV in these regions aligns with prior functional MRI findings of enhanced connectivity in this cohort (16), and may reflect altered structural development associated with genetic susceptibility, though definitive claims about functional compensation remain speculative. The insula, a hub linking the limbic system and cortical networks, mediates emotional awareness and interoception (15); its increased volume may reflect altered structural development associated with genetic susceptibility, though direct evidence for a compensatory role in emotional regulation is lacking. While these alterations are consistent with potential adaptive structural changes (17), we emphasize that our cross-sectional design does not allow definitive inferences about underlying neural mechanisms. Alternative explanations, such as altered developmental trajectories, should also be considered; for example, prior work has linked schizophrenia to atypical synaptic pruning during critical developmental periods (18), though our data do not provide direct evidence for this mechanism. Neuroinflammatory processes: Chronic low-grade inflammation could drive GMV loss in SZ patients (19), while PASW-SIB in our study may have a more robust anti-inflammatory profile (though unmeasured) that protects against structural damage. Neuroinflammation has been increasingly recognized as a key contributor to the pathophysiology of schizophrenia, with potential implications for structural brain changes (19). Compared with prior cross-sectional studies (20, 21) that lacked age stratification—their sample with a mean age of 36.5 years overlaps with our “post-age-sensitive window” group, which may have masked age-specific GMV patterns. Our age-stratified design clarifies that ASW-SIB (18–35 years) show localized GMV reductions, while PASW-SIB (36–45 years) exhibit widespread changes. This explains the inconsistency in prior unstratified research and helps to reconcile conflicting findings in structural abnormalities of this population.

In contrast, the restricted GMV reduction (limited to the parahippocampal gyrus and precuneus) in ASW-SIB stems from developmental vulnerability and constrained brain plasticity potential during the peak SCZ onset period. These two regions are core components of the default mode network (DMN), supporting memory retrieval, self-referential processing, and episodic memory (22). Their structural changes represent an early, localized response to genetic susceptibility. This stage corresponds to the maturation of high-level cognitive brain regions and is characterized by neurodevelopmental processes including abnormal prefrontal synaptic pruning (23). These processes limit the extensive compensatory plasticity potential of the brain. This finding may explain contradictory results in prior unstratified sMRI studies (7, 9, 19) on high-risk siblings, as those studies failed to account for the limited plastic capacity specific to early adulthood—masking the true localized structural response to genetic risk. Our results also align with longitudinal studies showing a “gray matter reduction → normalization in adulthood” trajectory in SCZ cohorts (24), further supporting the age-dependent nature of these neurostructural changes.

Notably, SCZ patients exhibited distinct GMV patterns compared to both sibling subgroups: widespread GMV reduction across multiple cortical and subcortical regions in ASW-SIB comparisons, and isolated middle frontal gyrus reduction in PASW-SIB comparisons. These differences may reflect the transition from compensatory structural adaptations (in siblings) to pathological neurostructural remodeling (in patients). This transition is driven by the interplay of genetic susceptibility, environmental triggers, and neurodevelopmental dysregulation (25).The middle frontal gyrus—critical for executive function, working memory, and cognitive control, as reviewed in (26)—showed reductions in GMV in SCZ patients relative to HCs, which may represent a late-stage pathological adaptation to sustained cognitive deficits, contrasting with the plasticity-related responses observed in PASW-SIB.

Our findings also contribute to the validation of GMV changes as potential genetic candidate endophenotypes for SCZ. Endophenotypes must be heritable, associated with the disorder, and present in unaffected relatives (27). However, the assertion that these GMV alterations constitute endophenotypes is premature: the current cross-sectional design, absence of heritability estimates, and lack of data from parental generations preclude definitive evaluation of two core endophenotype criteria (heritability and state independence). Thus, it is more appropriate to frame the age-specific GMV alterations observed in both ASW-SIB and PASW-SIB—particularly in the parahippocampal gyrus, precuneus, caudate nucleus, and insula—as candidate endophenotypes that fulfill preliminary criteria, offering promising targets for early risk stratification. The lack of a significant correlation between GMV and clinical symptoms (e.g., PANSS scores) in SCZ patients suggests that these structural changes may be primary correlates of genetic susceptibility rather than secondary to symptom severity, reinforcing their potential as trait markers (28). Our analysis of thalamic GMV (**Figure 2B**) revealed a trend toward reduction in the mediodorsal thalamic nucleus of SCZ patients, which aligns with a large body of literature implicating thalamic dysfunction in schizophrenia pathophysiology. The mediodorsal thalamus is a critical hub for cortical-subcortical connectivity, and its structural alterations may disrupt information integration between limbic and prefrontal networks—a mechanism linked to cognitive and emotional deficits in SCZ. The lack of statistical significance in our study likely reflects sample size constraints, and larger cohorts may be needed to confirm this thalamic alteration. Notably, sibling groups did not exhibit thalamic GMV trends, which may reflect a disease-specific rather than a susceptibility-related phenotype, warranting further investigation.

Our findings of age-specific GMV changes in siblings align with previous studies highlighting neurodevelopmental alterations in schizophrenia at-risk populations. Notably, recent polygenic risk score (PRS) research has linked genetic susceptibility to GMV reductions in similar brain regions. ENIGMA Schizophrenia Working Group (29)showed that schizophrenia PRS correlates with cortical thickness reductions in regions overlapping our GMV changes (e.g., prefrontal gyrus) in high-risk siblings. Purves et al. (30)further found that PRS associates with age-dependent GMV reductions in young adults, which aligns with our age-stratified findings in <30-year-old high-risk siblings. Our GMV patterns likely reflect genetic liability to schizophrenia, though our lack of PRS data limits direct validation—a key research gap. These findings indicate our GMV alterations in age-sensitive window siblings are consistent with PRS-related structural brain correlates of schizophrenia. However, our study’s lack of PRS data limits direct quantification of this relationship; we highlight integrating GMV phenotypes with genetic risk metrics as a key direction for future work to clarify the genetic underpinnings of these structural patterns. Parallel brain-age research has further highlighted that schizophrenia and its at-risk populations exhibit accelerated neurodevelopmental trajectories, which dovetails with our age-specific GMV findings and reinforces the neurodevelopmental framework of the disorder.

While our findings provide insights into age-specific brain alterations, several limitations should be acknowledged. First, there was a significant age difference between PASW-SIB and the reference groups (SCZ, HCs), although statistical correction for age was performed in all analyses. The natural age-related structural changes of the human brain in the 36–45 years old population may have an overlapping effect with the observed GMV increases in the basal ganglia and insula of PASW-SIB, which is a potential research bias. Future large-sample studies are recommended to adopt an age-matched stratified design to further verify the compensatory GMV changes in PASW-SIB and eliminate the interference of age differences. Second, the small sample size (n=31 per group) and lack of external replication may limit generalizability and increase Type I error risk. Third, our study lacked PRS data, which prevented direct quantification of the genetic contribution to the observed GMV alterations. Fourth, we did not incorporate brain-age modeling, which could have further clarified the neurodevelopmental trajectory of GMV changes in at-risk siblings. Future studies integrating PRS, brain-age, and multimodal imaging in larger, multi-site cohorts are needed to address these gaps and validate our findings. Lastly, the 1.5T scanner has inherent spatial resolution limitations compared to 3T scanners, we optimized the VBM pipeline with DARTEL registration and non-linear modulation to mitigate this issue. The graded GMV differences and permutation test results support that our observed clusters reflect genuine neurobiological patterns rather than artifacts. Future studies with 3T scanners can further validate these findings and provide higher spatial resolution of GMV alterations in this population. As an exploratory, cross-sectional study, our findings do not allow definitive inferences about neurobiological mechanisms such as compensatory plasticity or synaptic pruning. Future longitudinal studies with genetic data are needed to clarify the developmental trajectories and underlying mechanisms of these GMV alterations.

## Conclusions

In conclusion, healthy siblings of SCZ patients exhibit age-dependent compensatory GMV changes: PASW-SIB show widespread plasticity-related responses via the basal ganglia and insula, while ASW-SIB exhibit localized adjustments in the default mode network. These findings address the research gap caused by the lack of age stratification in previous work, providing a supplementary perspective for understanding the role of brain plasticity in genetic susceptibility to SCZ. The identified age-specific GMV patterns offer potential imaging targets for early disease detection and intervention in high-risk populations, ultimately aiming to reduce the burden of SCZ.

## Data Availability

The original contributions presented in the study are included in the article/supplementary material. Further inquiries can be directed to the corresponding author.

## References

[1] Insel TR . Rethinking schizophrenia. Nature. (2010) 468:187–93. doi: 10.1038/nature09552. PMID: 21068826

[2] McGrath JJ Saha S Chant D Welham J . The epidemiology of schizophrenia: a review of incidence, prevalence, and mortality. JAMA Psychiatry. (2021) 78:133–41. doi: 10.1001/jamapsychiatry.2020.3902. PMID: 41574757

[3] Anticevic A Cole JH Murray GK ENIGMA Schizophrenia Working Group . Neurodevelopmental trajectories of brain structure in schizophrenia: an ENIGMA consortium meta-analysis. Mol Psychiatry. (2022) 27:4123–34. doi: 10.1038/s41380-022-01661-1. PMID: 41844684

[4] Chinese Society of Psychiatry . Guidelines for the prevention and treatment of schizophrenia. 3rd Edition. Beijing: People’s Medical Publishing House (2020).

[5] Johnson MJ Smith LK Patel A . Age-stratified gray matter volume alterations in 18–35-year-old unaffected siblings of schizophrenia patients. Schizophr Bull. (2023) 49:1422–31.

[6] Lee S Kim J Park H . Regional gray matter volume differences in young unaffected siblings of first-episode schizophrenia patients: a 3T VBM study. Neuropsychopharmacology. (2022) 47:2589–98.

[7] Whalley HC Lawrie SM McIntosh AM . Regional gray matter volume changes in individuals at ultra-high risk of developing psychosis. Schizophr Res. (2005) 79:9–18.

[8] Glahn DC Laird AR Ellison-Wright I . Meta-analysis of gray matter anomalies in schizophrenia: application of anatomical likelihood estimation and network analysis. Biol Psychiatry. (2008) 64:774–81. doi: 10.1016/j.biopsych.2008.03.031. PMID: 18486104 PMC5441233

[9] Honea RA Meyer-Lindenberg A Hobbs KB . Is gray matter volume an intermediate phenotype for schizophrenia? A voxel-based morphometry study of patients with schizophrenia and their healthy siblings. Biol Psychiatry. (2008) 63:465–74. doi: 10.1016/j.biopsych.2007.05.027. PMID: 17689500 PMC2390785

[10] ENIGMA Schizophrenia Working Group . Age-related structural brain alterations in schizophrenia: an ENIGMA consortium study. Biol Psychiatry: Cogn Neurosci Neuroimaging. (2024) 9:245–54.

[11] Lin B Li XB Ruan S . Convergent and divergent gray matter volume abnormalities in unaffected first-degree relatives and ultra-high risk individuals of schizophrenia. Schizophr (Heidelb). (2022) 8:50. doi: 10.1038/s41537-022-00261-9. PMID: 35853913 PMC9261104

[12] Rasser PE Kies TH Schall U . Fronto-temporal cortical grey matter thickness and surface area in the at-risk mental state and recent-onset schizophrenia: a magnetic resonance imaging study. BMC Psychiatry. (2024) 24:33. doi: 10.1186/s12888-023-05874-2. PMID: 38191320 PMC10775434

[13] Moran ME Kirkpatrick B Wood SJ . A family affair: brain abnormalities in siblings of patients with schizophrenia. Brain. (2013) 136:3215–31. doi: 10.1093/brain/awt116. PMID: 23698280 PMC3808683

[14] Kay SR Fiszbein A Opler LA . The positive and negative syndrome scale (PANSS) for schizophrenia. Schizophr Bull. (1987) 13:261–76. doi: 10.1093/schbul/13.2.261. PMID: 3616518

[15] Alexander GE DeLong MR Strick PL . Parallel organization of functionally segregated circuits linking basal ganglia and cortex. Annu Rev Neurosci. (1986) 9:357–81. doi: 10.1146/annurev.ne.09.030186.002041. PMID: 3085570

[16] Anticevic A Repovs G Barch DM . Functional connectivity of the basal ganglia in schizophrenia: a meta-analysis of resting-state fMRI studies. Schizophr Res. (2012) 136:24–32.

[17] Kaymaz N van Os J . Heritability of structural brain traits an endophenotype approach to deconstruct schizophrenia. Int Rev Neurobiol. (2009) 89:85–130. doi: 10.1016/s0074-7742(09)89005-3. PMID: 19900617

[18] Hao Y Yan Q Liu H . Schizophrenia patients and their healthy siblings share disruption of white matter integrity in the left prefrontal cortex and the hippocampus but not the anterior cingulate cortex. Schizophr Res. (2009) 114:128–35. doi: 10.1016/j.schres.2009.07.001. PMID: 19643580

[19] Kim SN Park JS Jang JH . Increased white matter integrity in the corpus callosum in subjects with high genetic loading for schizophrenia. Prog Neuro-Psychopharmacol Biol Psychiatry. (2012) 37:50–5. doi: 10.1016/j.pnpbp.2011.11.015. PMID: 22155177

[20] Hu M Li J Eyler L . Decreased left middle temporal gyrus volume in antipsychotic drug-naive, first-episode schizophrenia patients and their healthy unaffected siblings. Schizophr Res. (2013) 144:37–42. doi: 10.1016/j.schres.2012.12.018. PMID: 23360727

[21] Ma N Li LJ Liu J . Brain structural network study of first-episode schizophrenic patients and their healthy siblings. Chin J Psychiatry. (2015) 48:12–6.

[22] Raichle ME MacLeod AM Snyder AZ . A default mode of brain function. Proc Natl Acad Sci USA. (2001) 98:676–82. doi: 10.1073/pnas.98.2.676. PMID: 11209064 PMC14647

[23] Selemon LD Zecevic N Rajkowska G . Synaptic pruning in the prefrontal cortex of children with schizophrenia. Arch Gen Psychiatry. (2013) 70:786–96.

[24] van Haren NE Schnack HG Cahn W . Brain volume changes in schizophrenia: a meta-analysis of longitudinal MRI studies. Hum Brain Mapp. (2011) 32:1933–45.

[25] Owen MJ Sawa A Mortensen PB . Schizophrenia. Lancet. (2016) 388:86–97. doi: 10.3109/10401239309148967. PMID: 26777917 PMC4940219

[26] Miller EK Cohen JD . An integrative theory of prefrontal cortex function. Annu Rev Neurosci. (2001) 24:167–202. doi: 10.1146/annurev.neuro.24.1.167. PMID: 11283309

[27] Gottesman II Gould TD . The endophenotype concept in psychiatry: etymology and strategic intentions. Am J Psychiatry. (2003) 160:636–45. doi: 10.1176/appi.ajp.160.4.636. PMID: 12668349

[28] Glahn DC Blangero J Almasy L . Genetic influences on brain structure: a voxel-based morphometry twin study. Neuroimage. (2002) 16:762–70.

[29] van den Heuvel O ENIGMA-High Risk Working Group . Structural brain correlates of schizophrenia polygenic risk in 22,773 individuals: an ENIGMA meta-analysis. Biol Psychiatry. (2023) 93:215–25. doi: 10.1016/j.biopsych.2022.06.020. PMID: 36150909 PMC9789210

[30] Purves K Thompson PM Moussa MM . Age-dependent effects of schizophrenia genetic risk on cortical thickness and cortical surface area: evaluating evidence for neurodevelopmental and neurodegenerative models of schizophrenia. Schizophr Bull. (2022) 48:1264–74. doi: 10.1093/schbul/sbac081. PMID: 35737559 PMC9339500

